# Comparative analysis of ARIMA and Holt-Winter’s additive models for describing human respiratory syncytial virus activity in Yaoundé, Cameroon

**DOI:** 10.3389/ijph.2026.1608524

**Published:** 2026-05-06

**Authors:** Moise Henri Moumbeket-Yifomnjou, Chavely Gwladys Monamele, Desmon Toutou Tsafack, Mohamadou Ripa Njankouo, Aristide Mounchili-Njifon, Paul Alain Tagnouokam-Ngoupo, Abdou Fatawou Modiyinji, Boyomo Onana, Richard Njouom

**Affiliations:** 1 Centre Pasteur du Cameroun, Yaoundé, Cameroon; 2 Laboratory of Microbiology, Department of Microbiology, Faculty of Sciences, University of Yaounde I, Yaoundé, Cameroon

**Keywords:** ARIMA model, Cameroon, Holt-Winter additive model, human respiratory syncytial virus, meteorological parameters

## Abstract

**Objectives:**

Human respiratory syncytial virus (HRSV) is a major cause of respiratory infections in children and older adults. This study compared the Autoregressive Integrative Moving Average (ARIMA) and Holt-Winter’s Additive models to describe HRSV activity in Yaoundé, Cameroon.

**Methods:**

In a three-year retrospective study (July 2020–December 2022), analyzed 1,774 nasopharyngeal samples from patients with severe acute respiratory infections (SARI) and influenza-like illness (ILI) were analysed across five sentinel sites in Yaoundé. The ARIMA model assessed the relationship between HRSV activity and meteorological factors (temperature, humidity, rainfall, solar radiation), while Holt-Winter’s Additive model described HRSV activity without climate variables. Model performance was evaluated using stationary R^2^ and root mean square error (RMSE).

**Results:**

HRSV was detected in 8.5% (151/1774) samples. Holt-Winter’s model outperformed ARIMA, achieving a stationary R^2^ of 77.6% and an RMSE of 7.40. ARIMA models for individual climate variables performed poorly (<6% R^2^), but the combined 12-variable model improved to 56.4% and an RMSE of 12.94.

**Conclusion:**

Holt-Winter’s model is more effective for predicting HRSV activity. These findings can guide public health interventions to reduce HRSV’s impact in Cameroon.

## Introduction

One of the most common viruses infecting children worldwide is the human respiratory syncytial virus (HRSV), which is also increasingly recognized as a serious infection in adults, particularly the older persons. In 2019, data from a systematic analysis showed that approximately 33 million episodes of HRSV-associated ALRI (Acute Lower Respiratory Infections) have resulted in 3.6 million hospital admissions for overall mortality of 101,400 deaths in children under 5 years of age, with more than half of severe episodes occurring in the first year of life [1]. During this first year of life, a second systematic review and meta-analysis estimated that in 2019, 1,650,000 episodes of HRSV-associated ALRI resulted in 533,000 hospitalizations of premature infants [2]. First identified in chimpanzees by Chanock and al., in 1956, HRSV is an enveloped, single-stranded RNA virus [3, 4]. Seasonal HRSV is caused by HRSV A and B viruses and their genotypes, which circulate worldwide.

Initial results from the World Health Organization’s (WHO’s) global surveillance of HRSV have shown that HRSV has different seasonal patterns in temperate and tropical regions, depending on the country [5]. In the tropics of both hemispheres, viral activity often begins in late summer, whereas in most temperate regions it begins in late autumn or early winter [1, 6]. Although seasonality is strongly associated with geographical parameters like latitude and longitude and meteorological parameters like temperature, humidity, and precipitation, these year-to-year variations in local HRSV seasonality remain largely unexplained [7–9].

However, research on the relationship between HRSV seasonality and meteorological factors in Cameroon is lacking or even non-existent. Furthermore, it is known that anticipating the dynamics of HRSV infection, which is responsible for a significant proportion of lower respiratory tract infections in people of all ages, particularly infants, young children, and the older persons, could be useful in assessing the epidemiological burden of HRSV. This anticipation could also help to plan the healthcare resources needed to treat severe respiratory infections caused by this virus in the region. In this study, we compared two-time series models: The Autoregressive Integrative Moving Average (ARIMA) and Holt-Winter’s additive model to describe laboratory-confirmed HRSV cases from 2020 to 2022 with changes in temperature, humidity, precipitation, and solar radiation.

## Methods

### Study location and setting

Cameroon’s sentinel influenza surveillance network has been operational since 2007. Like in many other countries, it aims to track the spread of influenza viruses and other respiratory viruses of public health importance, such as HRSV and, more recently, SARS-CoV-2. This surveillance system now includes 19 sites spread across all 10 administrative regions of the country. Some of these sites have an ILI (Influenza-Like Illness) surveillance program, some have SARI (Severe Acute Respiratory Infection) surveillance, and some have both.

**FIGURE 1:**
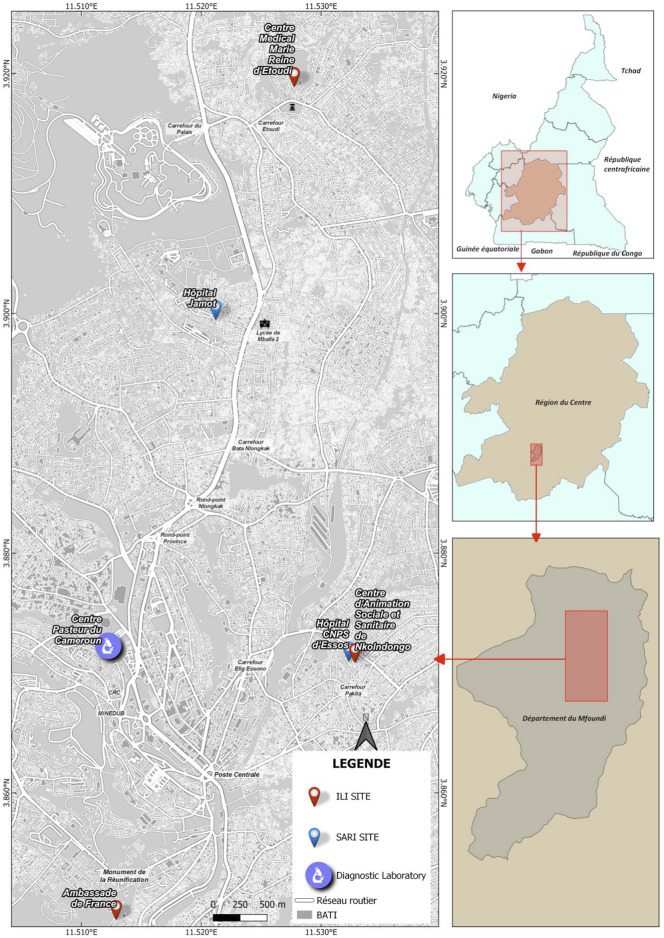
Map showing location of sentinel sites (Yaoundé, Cameroon, 2020–2022).

For this study, we used data collected at sentinel sites in Yaoundé, the capital of Cameroon, over 3 years (from July 2020 to December 2022). Yaoundé has a population of around 4.5 million and a tropical climate profile, albeit tempered by altitude, with four seasons: two rainy seasons and two dry seasons. The sites used in our analysis included two SARI sites, the “Centre Hospitalier d'Essos” and the “Hôpital Jamot”, as well as three ILI sites, including the “Centre Médico-Social Ambassade de France”, the “Centre d’Animation Sociale et Sanitaire de Nkolndongo” and the “Centre Médical d'Etoudi”. All laboratory analyses to confirm HRSV cases were performed at the Centre Pasteur du Cameroun (CPC) (Figure 1).

### Study subjects

All patients presenting with symptoms of ILI or SARI at the focal sites were recruited. As previously reported [10], the WHO case definition was used to identify cases of ILI or SARI. Patients of all ages with signs of ILI (an acute respiratory infection with fever measured at a temperature of 38 °C and cough occurring within the previous 10 days) or SARI (an acute respiratory infection with fever or fever measured at a temperature of 38 °C and cough occurring within the previous 10 days and requiring hospitalization) were, in brief, the site-level inclusion criteria [11].

### Virus detection

Each enrolled patient provided a nasopharyngeal and/or oropharyngeal sample. After collection, samples were preserved and transported to the virology department of the CPC for immediate processing or storage at 4 °C until analysis according to WHO guidelines [12]. All procedures for handling biological samples have been previously documented [10]. Briefly, nasopharyngeal swabs were tested for influenza A and B, HRSV, and SARS-CoV-2 using real-time RT-PCR assays.

### Climate data

Meteorological data considered in this study included earth surface temperature (EaST), the temperature at 2 m (T_2M_), wet bulb temperature at 2 m (WbT_2M_), specific humidity at 2 m (SH_2M_), relative humidity at 2 m (RH_2M_), dew/frost point at 2 m (DFPt_2M_), average corrected precipitation (AvCP), cumulative rainfall (CumRF), and solar radiation (SolRad). The variables were collected at monthly intervals from ground stations in the city of Yaoundé, located at latitude 3°50′29.04″N and longitude 11°29′31.56″, and downloaded from the National Aeronautics and Space Administration (NASA) database at https://power.larc.nasa.gov/data-access-viewer.

### Data analysis

Data on positive cases of HRSV for this study were obtained from 2020 to 2022. Monthly counts of positive cases were used as dependent variables, while monthly values of 12 climate variables were used as independent variables. These data were combined in an Excel spreadsheet and then imported into SPSS software version 26.0 (IBM, Chicago, IL, USA) for analysis. A comparative analysis of two time series models was used: the Autoregressive Integrated Moving Average (ARIMA) model and Holt-Winters’s additive model. The ARIMA model was used to examine the interactions between each climate variable and overall viral activity, whereas the additive Holt-Winter model, which is designed to capture seasonal patterns, does not account for climate variables.

ARIMA models are suitable for analyzing and forecasting time series data, based on three fundamental components: (1) autoregressive (AR) models, (2) integration (I), which determines the level of stationarity of the variable under study, and (3) the moving average (MA), which expresses the current value of the variable in question. The ARIMA model has three parameters: P, D, and Q. These are usually written as (p, d, q) (P, D, Q)m. In this notation, p and P refer to the autoregressive lags, q, and Q to the moving average lags, d and D to the differencing orders, and m to the seasonal cycle.

ARIMA models used the Box-Jenkins method. We used the Augmented Dickey-Fuller (ADF) test to see if the monthly HRSV case series was stable. Differencing was not necessary (d = 0, D = 0). The seasonal period was set at 12 months to match the annual cycle. Potential orders were determined through the application of autocorrelation function (ACF) and partial autocorrelation function (PACF) plots. The final model selection was then determined using the corrected Akaike Information Criterion (AICc) and the principle of parsimony. This approach led to the selection of an ARIMA (0,0,0) (0,0,0) model, which is characterized by a constant structure, for the multivariate model that included all twelve climate variables. As a result, the climate variables were used as direct predictors, without any additional autoregressive or moving average components.

The Winter’s additive seasonal model was used to describe whether the HRSV data showed a consistent seasonal pattern over time with no trend. This model has zero orders of autoregression, one order of differencing and seasonal differencing, and orders 1, p, and p + 1 of the moving average, where p is the number of periods in a seasonal interval and p equals 12 for monthly data. The smoothing parameters—level (α), trend (γ), and seasonality (δ)—were used in a maximum likelihood estimation to determine them.

The performance of the time series models was compared using graphs, stationary R^2^, and error metrics such as root mean square error (RMSE).

We carried out a final execution of the out-of-sample series test to evaluate the quality of the forecasts. First, we segmented the 30-month period, spanning from July 2020 to December 2022, into two distinct sets. The first part, covering 18 months from July 2020 to December 2021, was used to determine the model. The second part, on the other hand, constitutes a test set encompassing the last 12 months, from December 2021 to December 2022. This latter set is used to measure the accuracy of the formulated forecasts.

Supplementary Figure S2 also illustrates a flowchart that summarizes the entire analytical process, from data preprocessing to model training, followed by the evaluation of its performance.

## Results

### Description of the socio-demographic characteristics of the study population

Supplementary Table S1 shows the socio-demographic characteristics of the patients who participated in the study. All participants were residents of Yaoundé, and the year 2021 had a higher percentage of cases (57.0%) compared to other years. The 30-65 age group was the most represented, while the over-65 age group was the least represented, according to the population’s age distribution. Males and females represented 46.4% (823/1774) and 44.2% (784/1774) of the registered patients, respectively, while 9.4% (167/1774) were of unspecified sex. The HRSV positivity rate was 8.5%.

### Description of HRSV positivity rates

During the study, we observed a heterogeneous pattern of virus circulation from year to year. The temporal distribution of the virus is shown in Supplementary Figure S1. The months with the highest HRSV peaks were December 2020, June 2021, April 2022, and December 2022.

### Description of meteorological parameters

#### Temperature

**FIGURE 2:**
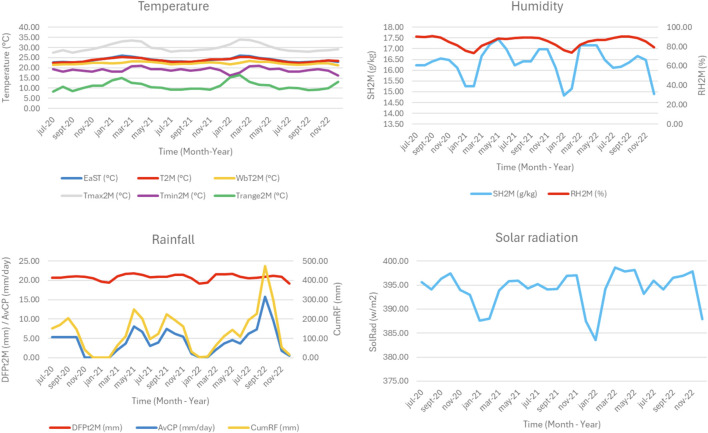
Temporal distribution of climate variables over the study period (Yaoundé, Cameroon, 2020–2022).

The descriptive statistics of the climatic parameters considered in our study and the HRSV cases for the Yaoundé city are summarized in Table 1. The data indicates that WbT2M (range 21.2 °C–23.3 °C, mean 22.2 °C) was the lowest temperature in the town, while T2M and EaST were the highest with mean values of 23.7 °C and 23.9 °C, respectively. Similar seasonal trends were observed for T2M and EaST, with peaks in January each year, while the lowest values were recorded between July and September. WbT2M showed a stable trend throughout the study period (Figure 2).

**TABLE 1:** Characteristics of the study variables (Yaoundé, Cameroon, 2020–2022).

Meteorological categories	Weather parameter	Minimum	Maximum	Mean	Standard error	Median	IQR
Temperature	EaST (°C)	22.69	26.13	23.91	0.1	23.68	[22.99; 24.48]
	T_2M_ (°C)	22.42	25.41	23.72	0.1	23.67	[22.83; 24.36]
	WbT_2M_ (°C)	21.25	23.37	22.25	0.1	22.27	[21.75; 22.58]
	Tmin_2M_ (°C)	16.20	20.91	18.90	0.2	19.01	[18.15; 19.28]
	Tmax_2M_ (°C)	27.49	34.00	29.91	0.3	29.19	[28.48; 31.65]
	Trange_2M_ (°C)	8.26	16.41	11.01	0.3	10.36	[9.46; 12.29]
Humidity	SH_2M_ (g/kg)	14.83	17.46	16.35	0.1	16.42	[16.11; 16.97]
	RH_2M_ (%)	73.44	90.75	85.11	0.9	86.72	[81.62; 89.2]
Rainfall	AvCP (mm)	0.00	15.79	4.16	0.6	3.85	[0.91; 6.26]
	CumRF (mm)	0.00	473.69	129.39	19.5	117.49	[39.73; 196.11]
	DFPt_2M_ (mm)	19.10	21.85	20.77	0.1	20.90	[20.58; 21.4]
Solar radiation	SolRad (w/m^2^)	383.58	398.66	394.19	0.6	394.77	[393.73; 396.90]

IQR, Interquartile range; EaST, earth surface temperature; T_2M_, temperature at 2 meters; WbT_2M_, wet bulb temperature at 2 meters; SH_2M_, specific humidity at 2 meters; RH_2M_, relative humidity at 2 meters; DFPt_2M_, dew/frost point at 2 meters; AvCP:,average corrected precipitation; CumRF, cumulative rainfall; SolRad, solar radiation.

#### Humidity

SH2M and RH2M showed a seasonal trend with lower values in February. The mean values for SH2M and RH2M were 16.3 g/kg (range: 14.8–17.5 g/kg) and 85.1% (range: 73.4–90.8), respectively.

#### Rainfall

Regarding rainfall parameters, the mean value for AvCP was 4.1 mm (range: 0–15.7 mm) and the average CumRF was 129.3 mm (range: 0–473.6 mm). Peaks occurred in May 2021 and September 2022 for both AvCP and CumRF. The months from December to February had almost no precipitation. DFPt2M, on the other hand, had a mean value of 20.8, showing very little variation over the study period.

#### Solar radiation

With a mean value of 394.1 w/m2, SolRad showed a fairly clear seasonal pattern, with seasonal lows in January and constant values between April and November.

### Description of the ARIMA model for HRSV activity

**FIGURE 3:**
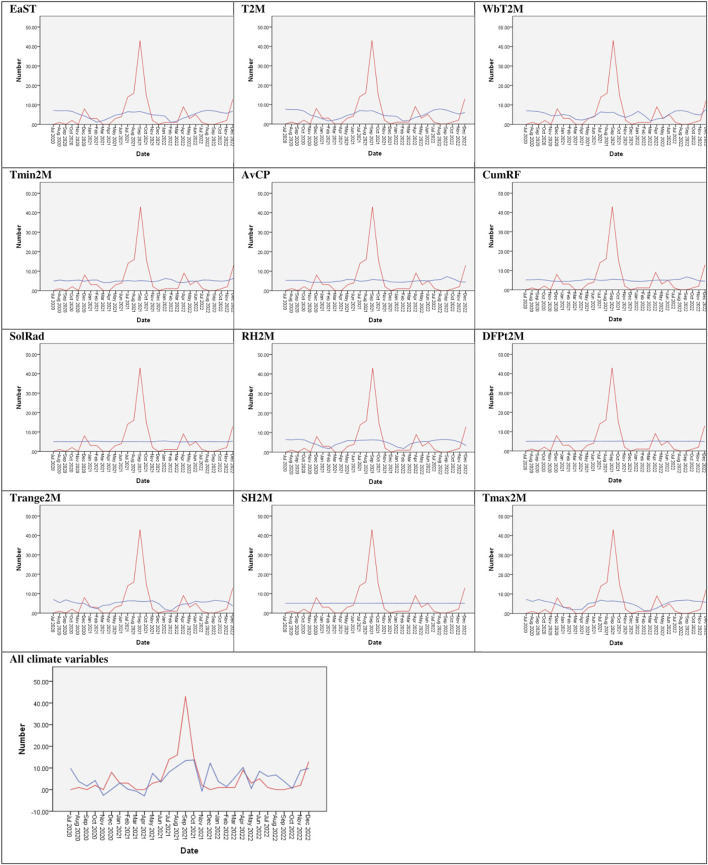
Performance of Autoregressive integrated moving average model with all climate variables (Yaoundé, Cameroon, 2020–2022). The x-axis shows months, the y-axis shows human respiratory syncytial virus cases. Observed values (•) and model fit (−) are shown. Seasonal peaks (December 2020, June 2021, April 2022, December 2022) are indicated by dashed lines.

No significant correlation (p < 0.05) was observed between individual climatological variables and HRSV activity (Table 2). With a RMSE of 12.94, the model incorporating the full set of climatological factors demonstrated superior performance, accounting for 56.4% of the variance in HRSV activity at this site. Figure 3 provides a graphical representation of the ARIMA models with all climate variables included as independent predictors in Yaoundé, Cameroon.

**TABLE 2:** Autoregressive integrated moving average model performance across all climate variables (Yaoundé, Cameroon, 2020–2022).

Model	Output metrics				Input variables			
	RMSE	Stat. R^2^	AIC	BIC	Detail	Climate variable	Estimate	P-value
ARIMA (0,0,0)(0,0,0)	8.601	0.048	-	-	Lag 0	EaST (°C)	−1.817	0.246
ARIMA (0,0,0)(0,0,0)	8.585	0.051	-	-	Lag 0	T_2M_ (°C)	−2.082	0.230
ARIMA (0,0,0)(0,0,0)	8.656	0.036	-	-	Lag 0	WbT_2M_ (°C)	−2.953	0.319
ARIMA (0,0,0)(0,0,0)	8.797	0.004	-	-	Lag 0	Tmin_2M_ (°C)	−0.466	0.750
ARIMA (0,0,0)(0,0,0)	8.787	0.006	-	-	Lag 0	AvCP (mm)	0.187	0.687
ARIMA (0,0,0)(0,0,0)	8.798	0.004	-	-	Lag 0	CumRF (mm)	0.005	0.755
ARIMA (0,0,0)(0,0,0)	8.813	0.000	-	-	Lag 0	SolRad (w/m^2^)	−0.032	0.943
ARIMA (0,0,0)(0,0,0)	8.686	0.029	-	-	Lag 0	RH_2M_ (%)	0.280	0.371
ARIMA (0,0,0)(0,0,0)	8.813	9.1, 10^−4^	-	-	Lag 0	DFPt_2M_ (mm)	0.113	0.960
ARIMA (0,0,0)(0,0,0)	8.685	0.029	-	-	Lag 0	Trange_2M_ (°C)	−0.712	0.370
ARIMA (0,0,0)(0,0,0)	8.813	9.4, 10^−7^	-	-	Lag 0	SH_2M_ (g/kg)	0.012	0.996
ARIMA (0,0,0)(0,0,0)	8.619	0.044	-	-	Lag 0	Tmax_2M_ (°C)	−0.902	0.268
ARIMA (0,0,0)(0,0,0)	12.94	0.564	146.21	157.78	Lag 0	All	21.824	0.240

Fit, fitting results; RMSE, Root mean square error; Stat.R^2^, Stationary R^2^; Coef., coefficient; AIC, Akaike Information Criterion; BIC, Bayesian Information Criterion; Tmin_2M_, minimum temperature at 2 meters; AvCP, average corrected precipitation; CumRF, cumulative rainfall; EaST, earth surface temperature; T_2M_, temperature at 2 meters; WbT_2M_, wet bulb temperature at 2 meters; SH2M, specific humidity at 2 meters; DFPt2M, dew/frost point at 2 meters; SolRad, solar radiation.

### Description of the Holt-Winter’s additive model for HRSV activity

**FIGURE 4:**
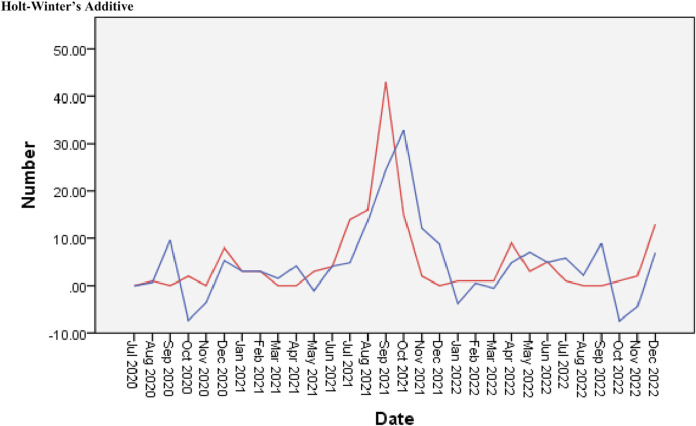
Performance of Holt-Winter’s Additive model with all climate variables (Yaoundé, Cameroon, 2020–2022). The x-axis shows months, the y-axis shows human respiratory syncytial virus cases. Observed values (•) and model fit (−) are shown. Seasonal peaks are marked with dashed lines.

The Holt-Winter’s Additive model was the best model for HRSV activity, explaining 72.6% of HRSV activity (Supplementary Table S2). Furthermore, a lower RMSE value for the Winters Additive model (7.289) indicates a better performance of this model in monitoring the spread of HRSV. In addition, a statistically significant correlation (P < 0.001) was observed between seasonal changes and HRSV activity. Figure 4 shows the graphical representation of the ARIMA models with all climate variables as independent predictors in Yaoundé, Cameroon.

### Out-of-sample validation

To assess the generalization capacity of the forecasts, both models were employed to estimate the number of HRSV infection cases over a 12-month observation period. In this context, the additive Holt-Winters model demonstrated superior effectiveness compared to the ARIMA model in terms of predictive performance. The RMSE associated with the Holt-Winters model was 20.50, whereas that of the ARIMA model was 27.07, as shown in Supplementary Table S3. These results indicate that the seasonal model can predict HRSV activity in this study with high accuracy, its error rate being lower by 24%.

## Discussion

This study aimed to compare two different models to describe HRSV activity in Yaoundé, Cameroon: the ARIMA model (with climatic variables) and the Winter’s additive model (without climatic variables). The data from 2020 to 2022 show heterogeneity of the HRSV circulation from year to year. Peaks were observed in December 2020, June 2021, April 2022, and December 2022. Sites in the same climatic region as ours have reported seasonal HRSV trends that are largely identical. The primary annual patterns of HRSV activity in these tropical and subtropical locations have been erratic, with extended HRSV circulations often lasting more than 6 months and highly variable seasonal onset and/or peak dates [5, 10, 13, 14]. This contrasts with temperate countries, where most data document HRSV activity with mostly annual patterns starting in winter and shorter HRSV season durations than documented in tropical and subtropical locations [9].

The observed variability suggests weather conditions may influence HRSV seasonality. This study found no correlation between any of the climatological factors and HRSV activity in Yaoundé. Haven used either several parameters for temperature, humidity, and rainfall each described HRSV activity very poorly (<6%). These results contrast with those from China, Kenya, and central Malaysia [15–17], which show a substantial correlation between HRSV activity and at least one of the variables. However, our findings are consistent with those of several other studies conducted in countries such as South Africa, Madagascar, and Mexico [18–20], which have indicated that climatic factors account for only a small proportion of the variability in HRSV activity. Possible reasons for these phenomena include variation in the timing of HRSV epidemics between countries, seasonal variation at the national and even sub-national level, tropical rainfall regimes, humidity cycles, the underlying mechanisms of climatic impacts, and non-climatic factors such as socio-behavioral determinants. Nevertheless, the ARIMA model considering all climate variables together showed an improved performance, explaining about 56.4% of HRSV activity in the capital city of Yaoundé. The observed synergistic effect of the model consisting of all climate variables suggests that integrating other cofounding factors such as age, holiday period and different human contact patterns in different climates may improve model performance [21].

In contrast, the Holt-Winters additive model provided a better explanation of HRSV activity, with a performance of 77.6%. This model could be useful for predicting future HRSV activity. The out-of-sample validation performed in this study further supported this hypothesis. The results allowed us to provide further significant evidence of the predictive utility of this model, as hardly any studies have explored a seasonal model to describe HRSV activity, since seasonality is generally considered to depend on climatological factors. Most previous studies have used either an ARIMA model [15, 22], logistic regression [23], a generalized linear time series model [24], or negative binomial regression analysis [21] to explore the association between HRSV activity and meteorological factors. It is unclear why the seasonal model outperformed the ARIMA model by such a wide margin. A model that incorporates both seasonal aspects and meteorological variables would be useful for improving the prediction of HRSV cases. While both models achieved a reasonable fit on the training set with an RMSE of 7.40 for Holt-Winters versus 12.94 for ARIMA, the seasonal model demonstrated much better generalization to unseen data, with a prediction error 24% lower on the test set.

This study had some important limitations. Although helpful, analysis of future trends in HRSV prevalence may not be definitive due to dynamic changes in social and environmental elements such as income, holiday seasons, COVID-19 restriction measures, and global warming. Predicting infection rates that take into account the co-circulation of other respiratory viruses is equally challenging. In addition, the surveillance data that we used for our analysis was not consistent throughout the year and was not representative of all ten regions of the country. The time series models can be sufficiently refined to provide reliable annual forecasts of the onset and peak of HRSV, which would undoubtedly help public health officials to anticipate and respond to HRSV epidemics. A larger and more reliable epidemiological dataset, along with more frequent environmental data collection (e.g., daily or weekly rather than monthly), and hybrid modeling approaches that combine mechanistic epidemic equations with machine learning—as has already been reported in some recent studies [25–27]—are examples of improvements that could enable a more accurate description and prediction of HRSV activity, while accounting for geographical variability.

## Supplementary material

SUPPLEMENTARY FIGURE S1 Human respiratory syncytial virus positivity rate in Yaoundé, Cameroon, 2020-2022.

SUPPLEMENTARY FIGURE S2 Flowchart of the analysis methodology.
