# Supplementary material for: Comparative analysis of ARIMA and Holt-Winter’s additive models for describing human respiratory syncytial virus activity in Yaoundé, Cameroon
Source: Int J Public Health. 2026 May 6;71:1608524. doi: 10.3389/ijph.2026.1608524 (PMC13186711; doi:10.3389/ijph.2026.1608524)
Supplement: Supplementary file 1 [file DataSheet1.zip › Supplementary material revised/Supplementary Table S1.docx]

**Supplementary Table S1:** Description of study population (Yaoundé, Cameroon, 2020 - 2022)

| **Characteristic** | **Sub-categories** | Overall **N (%)** |
| --- | --- | --- |
| Year | 2020 | 154 (8.7) |
|  | 2021 | **1011 (57.0)** |
|  | 2022 | 609 (34.3) |
| Age | <1 | 283 (16.0) |
|  | [1–5[ | 396 (22.3) |
|  | [5-15[ | 132 (7.4) |
|  | [15-30[ | 190 (10.7) |
|  | [30-65] | **502 (28.3)** |
|  | >65 | 95 (5.4) |
|  | Missing | 176 (9.9) |
| Sex | Male | **823 (46.4)** |
|  | Female | 784 (44.2) |
|  | Missing | 167 (9.4) |
| Source | Influenza surveillance | **1111 (62.6)** |
|  | SARS-CoV-2 surveillance | 167 (37.4) |
| HRSV results | Positive | 151 (8.5) |
|  | Negative | **1623 (91.5)** |
| Total |  | **1774** |

Each subcategory's most prevalent categories are indicated in **bold.**
