# Supplementary material for: Comparative analysis of ARIMA and Holt-Winter’s additive models for describing human respiratory syncytial virus activity in Yaoundé, Cameroon
Source: Int J Public Health. 2026 May 6;71:1608524. doi: 10.3389/ijph.2026.1608524 (PMC13186711; doi:10.3389/ijph.2026.1608524)
Supplement: Supplementary file 1 [file DataSheet1.zip › Supplementary material revised/Supplementary Table S2.docx]

**Supplementary Table S2:** Holt-Winter’s additive model performance with no climate predictors (Yaoundé, Cameroon, 2020 - 2022)

|  | **Fit** | | | | **Model parameters** | | | | |
| --- | --- | --- | --- | --- | --- | --- | --- | --- | --- |
|  | **RMSE** | **Stat. R^2^** | **AIC** | **BIC** | **α** | **γ** | **δ** | **Ljung-Box p-value** | **P-value** |
| Holt-Winter’s additive | 7.40 | 0.776 | 125.87 | 128.54 | 0.116 | 0.000 | 0.001 | < 0.01 | < 0.01 |

Fit: fitting results, RMSE = Root mean square error, Stat.R^2^ = Stationary R^2^, AIC = Akaike Information Criterion, BIC = Bayesian Information Criterion, α = level smoothing parameter; γ = trend smoothing parameter; δ = seasonal smoothing parameter.
