# Supplementary material for: Comparative analysis of ARIMA and Holt-Winter’s additive models for describing human respiratory syncytial virus activity in Yaoundé, Cameroon
Source: Int J Public Health. 2026 May 6;71:1608524. doi: 10.3389/ijph.2026.1608524 (PMC13186711; doi:10.3389/ijph.2026.1608524)
Supplement: Supplementary file 1 [file DataSheet1.zip › Supplementary material revised/Supplementary Table S3.docx]

**Supplementary Table S3:** Out-of-sample predictive performance of ARIMA and Holt-Winters additive models (Yaoundé, Cameroon, 2020-2022)

| Model | Training set (July 2020 – Dec 2021) | Test set (Jan 2022 – Dec 2022) |
| --- | --- | --- |
|  | **RMSE (fit)** | **RMSE (prediction)** |
| ARIMA (12 climate variables) | 12.94 | 27.07 |
| Holt-Winters additive | 7.40 | 20.50 |

RMSE = Root mean square error
