# Supplementary figures and images for: Comparative analysis of ARIMA and Holt-Winter’s additive models for describing human respiratory syncytial virus activity in Yaoundé, Cameroon
Source: Int J Public Health. 2026 May 6;71:1608524. doi: 10.3389/ijph.2026.1608524 (PMC13186711; doi:10.3389/ijph.2026.1608524)

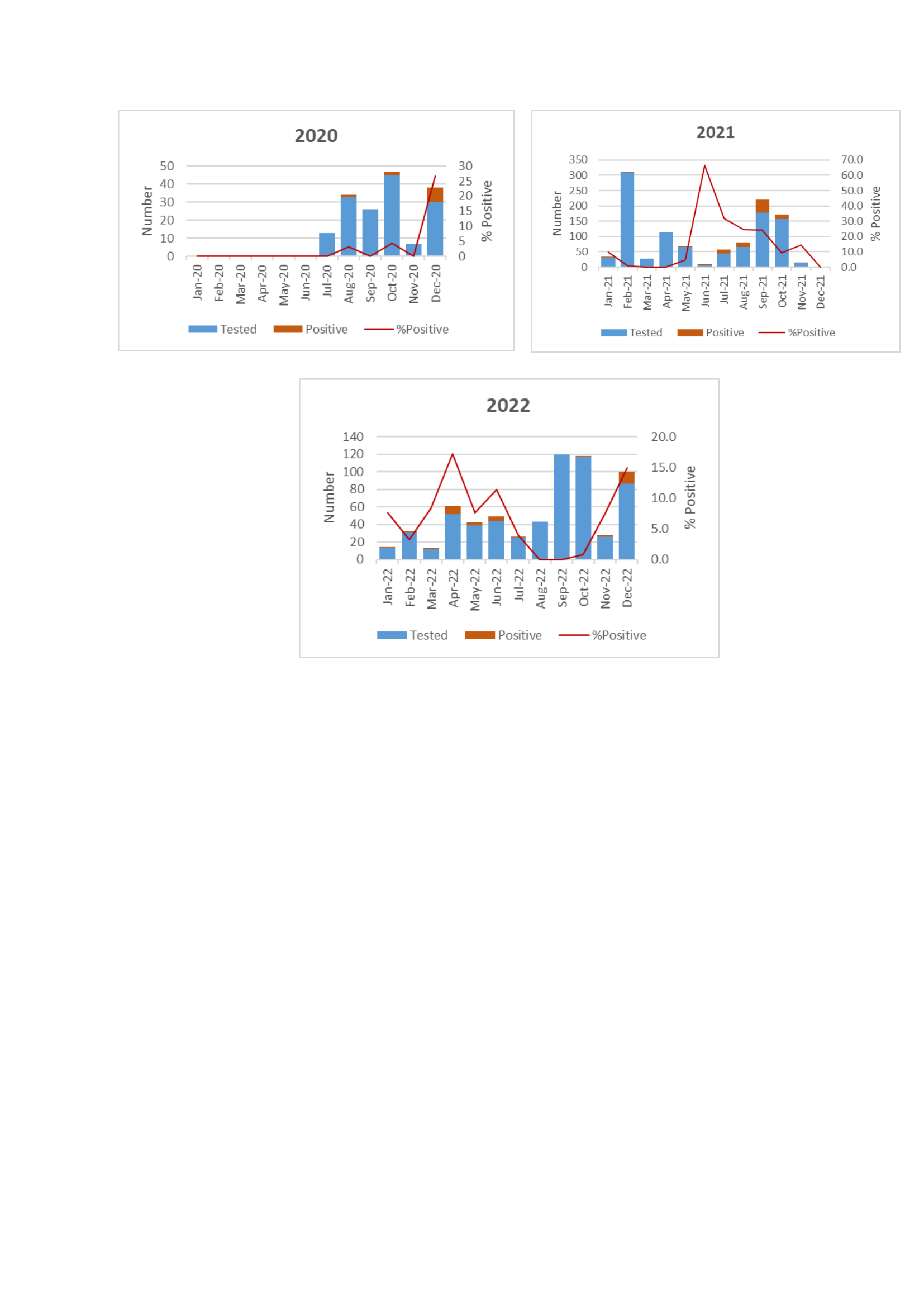

Supplement: Supplementary file 1 [file DataSheet1.zip › Supplementary material revised/Supplementary Figure S1.tiff]

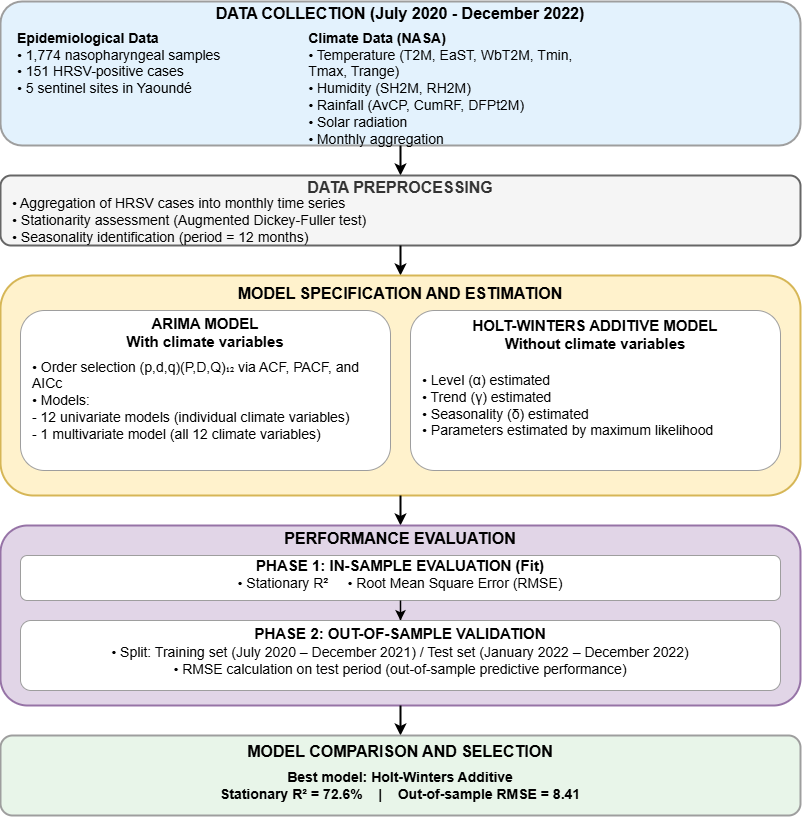

Supplement: Supplementary file 1 [file DataSheet1.zip › Supplementary material revised/Supplementary Figure S2.tiff]
